# Ethnobotanical Knowledge, Cultural Resilience, and the Sustainability of *Canthium lallanii* in Eshowe, South Africa

**DOI:** 10.3390/foods15203703

**Published:** 2026-10-17

**Authors:** Bahle N. Msomi, Khanyisile Sangcozi, Nontuthuko R. Ntuli, Noluthando N. Nkosi, Zoliswa Mbhele

**Affiliations:** Department of Botany, Faculty of Science, Agriculture, and Engineering, University of Zululand, KwaDlangezwa 3886, South Africa; bahlemsomi93@gmail.com (B.N.M.); sangcozikhanyisile06@gmail.com (K.S.); ntulir@unizulu.ac.za (N.R.N.); nkosinn@unizulu.ac.za (N.N.N.)

**Keywords:** wild edible fruits, umBhembethu, indigenous knowledge, ethnobotanical uses, food sovereignty, traditional food systems, intergenerational knowledge transmission, ethnobotanical indices, fermented beverages

## Abstract

Traditional food knowledge is a cornerstone of community resilience, cultural identity, and food security, yet it is increasingly threatened worldwide by habitat loss, land-use change, and urbanization, which both clears wild food habitats and draws younger generations away from the land-based practices through which such knowledge is learned and transmitted. *Canthium lallanii* R.Kr.Singh (syn. *Canthium spinosum* (Klotzsch ex Eckl. & Zeyh) Kuntze) is an underutilized indigenous fruit plant whose fruit is consumed as a snack and whose various parts are used in folk medicine, construction, and craft-making. However, it is still harvested exclusively from the wild and is vulnerable to local extinction. This study investigated the ethnobotanical uses of *C. lallanii* among three rural communities in Eshowe, South Africa, to document how traditional plant knowledge continues to underpin household nutrition, income generation, and cultural practice in the face of mounting environmental and social pressure. Data were collected through structured interviews with 100 randomly selected homesteads. The frequency index (*FI*), relative frequency of citation (*RFC*), use value (*UV*), and informant consensus factor (*ICF*) were calculated, and thematic analysis was used to interpret qualitative responses. Results showed that *C. lallanii* fruit is universally recognized and consumed as a snack (*FI* = 100%) and used to flavour traditional beer, while the plant is also drawn upon to treat conditions such as stomachache, pimples or acne, diabetes, and toothache, and for body-cleansing practices. Informant consensus was high for food (*ICF* = 0.99) and material uses (*ICF* = 0.91) but low for medicinal uses (*ICF* = 0.20), indicating that medicinal knowledge is held by only a few individuals. The branches and stems of the species support firewood, homestead construction, fencing, kraals, and craft production, activities that generate supplementary household income. Participants identified climate change, overharvesting, urbanization, deforestation, and the natural ageing of remaining stands as converging threats to the species’ survival. In response, community members articulated locally grounded conservation strategies, such as domestication, homestead cultivation, and the deliberate transmission of knowledge to younger generations. These strategies reflect community-driven efforts toward local control over food resources and long-term resource autonomy, consistent with food sovereignty principles. The findings highlight the value of documenting such knowledge before it is lost, and contribute case-based, empirical evidence to wider debates on how traditional plant-based food systems sustain autonomy, dignity, and cultural continuity for rural communities facing environmental and social change.

## 1. Introduction

Traditional food knowledge—the orally transmitted understanding of which plants to gather, how to prepare them, and when they become available—has long underpinned the resilience, cultural identity, and food security of rural and indigenous communities across diverse ecological and political landscapes [1,2]. This knowledge is rarely only nutritional; it is embedded in ritual, kinship, gendered labour, and local economies, and it is reproduced across generations through practice rather than formal instruction [3]. Globally, however, such knowledge systems are under sustained pressure from rapid socio-environmental change, land dispossession, the spread of industrial agriculture, and the cultural homogenization that accompanies urbanization [4]. Urbanization erodes traditional food knowledge through two linked pathways. First, the expansion of housing, roads, and commercial land clears the wild habitats in which food plants grow, removing the resource itself. Second, migration to towns for schooling and wage employment, together with growing reliance on purchased foods, reduces the time that younger people spend gathering, preparing, and eating wild foods alongside elders. Because such knowledge is learned by doing rather than through formal instruction, fewer opportunities for shared practice translate directly into fewer knowledge holders in the next generation [5,6]. These pressures fall disproportionately on communities already navigating marginalization [4]. At the same time, growing attention to food sovereignty, broadly defined as the right of local peoples to control their own food systems, markets, ecological resources, and food culture [7]. Moreover, decolonial approaches to food systems [8] has renewed interest in traditional foodways, not as nostalgic relics but as active, adaptive strategies through which communities sustain nutrition and livelihoods while resisting systemic marginalization, safeguarding biodiversity, and maintaining intergenerational continuity [9]. Community-based, plant-centred food sovereignty is often enacted through everyday practices at household and community levels, long before it is formally recognized in policy discourse. Ethnobotanical documentation provides a systematic means of making these often-overlooked food practices, plant knowledge and local management strategies visible [10,11,12].

However, despite this renewed interest, empirical, community-grounded documentation of traditional food knowledge remains comparatively scarce for many underutilized southern African indigenous fruit species [13]. *Canthium lallanii* R.Kr.Singh (syn. *Canthium spinosum* (Klotzsch ex Eckl. & Zeyh) Kuntze) is one such species. Although its consumption as a snack and its use in folk medicine have been briefly identified in earlier regional ethnobotanical surveys, no dedicated study has systematically quantified its uses, socio-economic significance, or the specific pressures threatening its persistence. This gap is significant precisely because species in this position of being locally valued but externally overlooked are those most likely to disappear. Their loss entails the disappearance of associated traditional knowledge before it can be formally documented. This study addresses this gap directly.

*Canthium lallanii*, commonly known as umBhembethu (isiZulu), belongs to the Rubiaceae family [14,15]. The species has long been known as *C. spinosum* (Klotzsch ex Eckl. & Zeyh.) Kuntze; however, that name is an illegitimate later homonym of *C. spinosum* (Thunb.) J.St.Hil. (a synonym of *Catunaregam spinosa*), and the replacement name *C. lallanii* R.Kr.Singh was therefore published in 2024 [16]. The earlier name is retained in the literature cited here, which predates this change. It is a spiny, multi-stemmed shrub or small tree, typically up to 4 m tall, with clustered leaves on short side shoots below each spine near the branch tips [14,17], and it occurs widely across Kenya, Mozambique, South Africa, Tanzania, and Zimbabwe [16]. In southern Africa, *C. lallanii* fruits are traditionally consumed as snacks, highlighting their nutritional and cultural significance within local food systems [15,17,18]. The ripe fruit is palatable, with a taste likened to that of dried peach [17], yet its nutritional and phytochemical composition has not been characterized. Beyond direct nutritional use, the species carries notable ethnomedicinal value. In KwaZulu-Natal and Eswatini, for example, a leaf infusion is traditionally used to treat stomach problems and diarrhea [15]. These practices are part of a broader body of traditional plant knowledge passed down through generations, an area of study called ethnobotany [19,20].

Ethnobotany, as the scientific study of how humans interact with plants for food, medicine, cultural expression, and ecological management [17,19,21], offers a direct lens into traditional food systems. Indigenous fruits occupy a particularly important place within these systems across African local communities, where they are frequently eaten raw as snacks and processed into secondary products that extend their nutritional and economic value [22]. Despite this centrality to everyday life, they remain broadly undervalued in mainstream food and conservation policy. They also face compounding threats from habitat loss, land-use change, modernization, and the erosion of the traditional knowledge systems that sustain their use [22,23]. Multiple plant parts beyond the fruit are drawn upon in traditional medicine to treat a wide range of ailments [24,25]. Indigenous fruit plants more broadly contribute to rural livelihoods and household resilience by supplying fuelwood, fibres, shelter materials, and other bio-based utilities [22]. It is these resources that carry weight for households navigating economic precarity or limited access to formal markets.

Documenting this knowledge therefore contributes to safeguarding community autonomy and dignity in the face of ongoing dispossession and cultural erosion. Ethnobotanical research plays a crucial role in recording traditional plant knowledge, guiding locally appropriate conservation priorities, and informing sustainable domestication and integration strategies that keep decision-making rooted in community practice rather than external prescription [26]. Because this knowledge is held disproportionately by older generations and transmitted informally, it is acutely vulnerable to disruption whenever habitat is lost, land use changes, or younger community members are drawn away from customary practices [22,23]. Ethnobotanical studies are consequently vital not only for recording traditional plant use but for validating and actively revitalizing it as a living resource for present and future food security [22].

Therefore, this study examines the ethnobotanical uses, socio-economic significance, and conservation status of *C. lallanii* among three rural communities in Eshowe, KwaZulu-Natal, South Africa. Specifically, the study aimed to: (i) document and quantify the food, medicinal, and material uses of *C. lallanii* using quantitative ethnobotanical indices (*FI*, *RFC*, *UV*, and *ICF*); (ii) identify which plant parts are used, by whom, and for what purposes; (iii) establish local perceptions of the drivers behind the species’ declining availability; (iv) record community-generated strategies for its conservation and the transmission of associated knowledge; and (v) compare these uses with those recorded among other cultural groups. In doing so, the study contributes empirical, community-grounded evidence to broader debates on how traditional plant-based food systems support autonomy, cultural continuity, and resilience for Indigenous and local communities confronting environmental and social change.

## 2. Materials and Methods

### 2.1. Study Area

**Figure 1:**
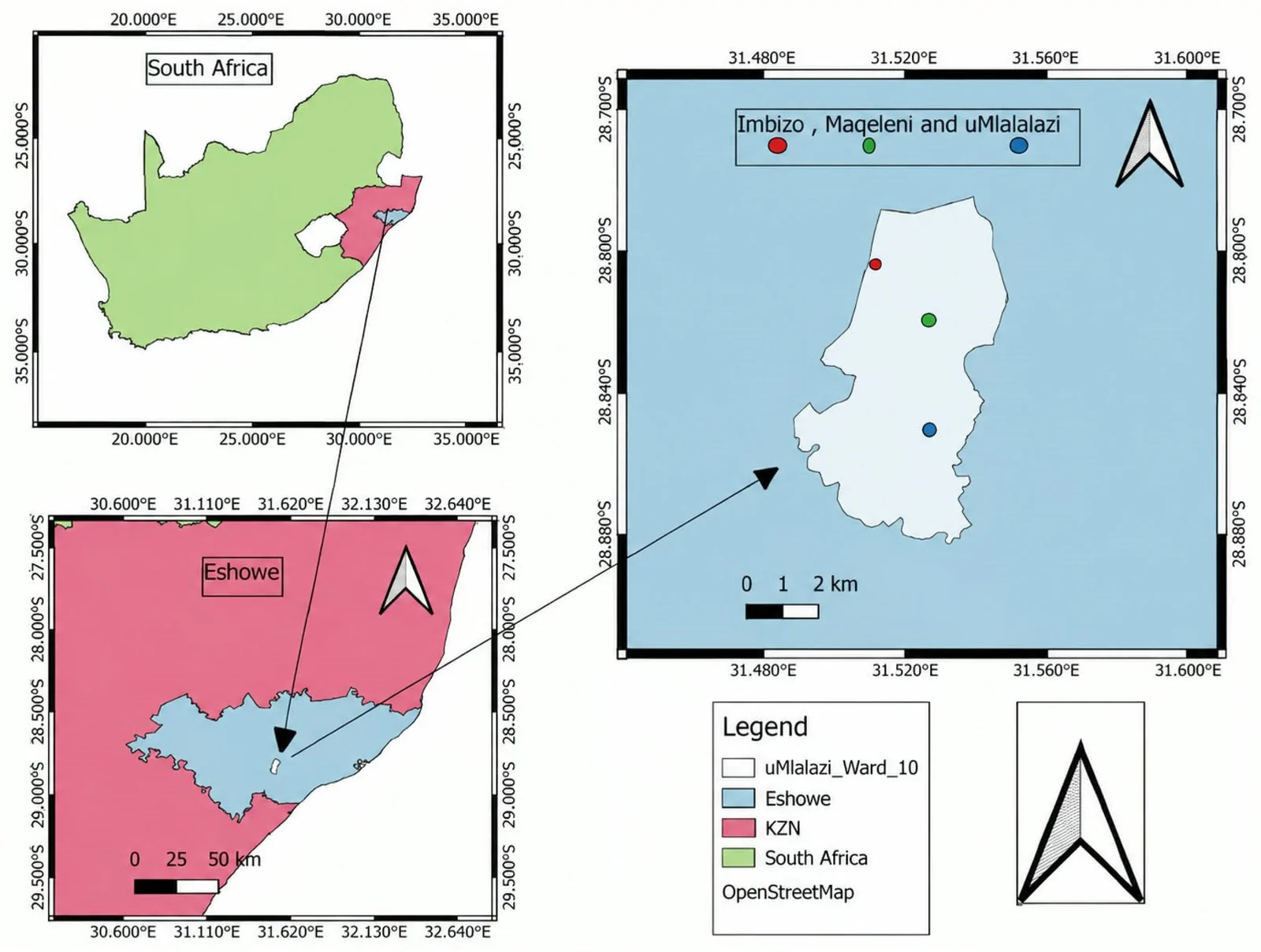
Eshowe in KwaZulu-Natal within the uMlalazi Local Municipality in the King Cetshwayo District, South Africa. Imbizo (red), Maqeleni (green), and uMlalazi (blue). Map prepared by Sangcozi Khanyisile (second author).

The study was conducted within three communities of Eshowe, namely Imbizo (27.7646° S, 31.5230° E), Maqeleni (28.7839° S, 31.5365° E), and UMlalazi (28.8216° S, 31.5370° E), located in the northern coastal region of KwaZulu-Natal Province, South Africa [27] (Figure 1). The vegetation type in this area is classified as Indian Ocean Coastal Belt, characterized by a blend of natural forests and subtropical vegetation [28]. Eshowe lies within the uMlalazi Local Municipality in the King Cetshwayo District and covers approximately 617.70 hectares of agricultural land [29]. Eshowe is a historically significant town, one of the oldest colonial settlements in Zululand. The landscape is dominated by rolling hills and lush forests, providing a diverse and ecologically important environment [27]. The climate is subtropical, with warm, wet summers and mild, dry winters. Annual rainfall ranges between 800 and 1200 mm, primarily occurring between October and March, while temperatures vary from 10 °C in winter to over 30 °C in summer [30]. These climatic conditions support a wide range of plant species, including indigenous forests that are a defining feature of the area. Eshowe’s rich biodiversity includes numerous plant species, some of which are endemic to the region. The diversity of plant life in the surrounding forested areas makes it an essential site for ecological research and conservation efforts [28].

Demographically, the uMlalazi Local Municipality had a population of 241,416 people in 45,119 households at the 2022 national census, of whom 95.7% identified as Black African [31]. IsiZulu is the dominant home language, spoken by approximately 92% of residents, and about 80% of the municipal population lives in traditional (tribal) authority areas, with 14% in urban areas and 6% on farms [32]. Livelihoods are strongly land-based: nearly half of all households (21,995 of 45,062) were classified as agricultural households in 2011, and the unemployment rate was 35.2% (45.1% among youth) [32].

### 2.2. Data Collection

A formal letter was addressed to the community leadership to request access to the area and permission to interact with residents. A preliminary field visit was undertaken within three communities of Eshowe, namely Imbizo, Maqeleni, and UMlalazi, to verify the presence of *C. lallanii* in the area and to introduce the objectives of the research project. Simple random sampling was used, a technique in which everyone in the population has an equal opportunity to be selected for the sample [33,34]. Indigenous knowledge on the ethnobotanical uses, socio-economic significance, conservation, and sustainability of *C. lallanii* was gathered from residents of 100 randomly selected homesteads through formal surveys. Before participation, everyone was asked to sign a consent form confirming their voluntary involvement. The structured interviews were based on a questionnaire comprising seven sections: (1) socio-demographic data, (2) traditional uses of *C. lallanii*, (3) medicinal uses and preparation methods, (4) food uses and preparation methods, (5) socio-economic importance, (6) conservation and sustainability concerns, and (7) additional information. Questions on plant uses were open-ended: participants were asked to list, from free recall, every use of the species that they knew, without being prompted with a predefined list of uses, and then to describe the plant part used and the method of preparation and administration for each use reported. Data were collected between (January 2025) and (April 2025). All interviews and discussions were conducted in isiZulu to ensure clear and effective communication with participants and to allow knowledge to be expressed in participants’ own cultural and linguistic frame of reference.

### 2.3. Data Analysis

The frequency index (*FI*) was calculated using Equation (1):

(1) $$\mathrm{FI}=\frac{\mathrm{FC}}{N}\times 100$$

for the 100 participants who identified ethnobotanical uses of *C. lallanii*, where *FC* is the number of participants who mentioned a particular use, and *N* is the total number of participants [35]. Qualitative data from interviews were analyzed using thematic analysis, with transcripts systematically coded to identify and interpret key themes related to the ethnobotanical uses, socio-economic significance, and conservation of *C. lallanii* [36]. Because each participant could report a given use only once, *FI* is the relative frequency of citation expressed as a percentage; the relative frequency of citation (*RFC*) [37] therefore equals *FI*/100 and ranges from 0 (no participant reports the use) to 1 (all participants report it). To capture the overall and category-level importance of the species, the use value (*UV*) [38] and the informant consensus factor (*ICF*) [39,40] were also calculated:

(2) $$\mathrm{RFC}=\frac{\mathrm{FC}}{N}$$

(3) $$\mathrm{UV}=\frac{\sum U_{i}}{N}$$

(4) $$\mathrm{ICF}=\frac{N_{\mathrm{ur}}-N_{t}}{N_{\mathrm{ur}}-1}$$

where *Ui* is the number of uses reported by a participant, *N* is the total number of participants (100), *Nur* is the number of use reports in a use category, and Nt is the number of distinct uses reported in that category. Because this study concerns a single species, *ICF* was adapted by treating distinct uses, rather than taxa, as the units counted in *Nt*. *ICF* ranges from 0 to 1, with values close to 1 indicating that participants agree on the uses within a category. *UV* was calculated for the species overall and for each use category (food, medicine, and other/material uses).

The indices were chosen to fit the design of the study, which documents the uses of a single species recorded by free recall from 100 randomly selected participants. *FI*, and its proportional form *RFC*, show how widely each individual use is known [37]; *UV* summarizes the overall and category-level importance of the species from the number of use reports [37,38]; and *ICF* shows whether knowledge within a use category is widely shared or held by only a few people [39,40], which bears directly on knowledge transmission (objectives (ii) and (iv)). Together, these measures answer the questions posed in objective (i): how widely each use is known, how much each use category contributes, and how far participants agree. Other common indices were considered but not used, because they are designed to compare several species or need data that this study did not collect. The fidelity level (*FL*) gives the proportion of informants citing a species who cite it for a particular use [41]; because all 100 participants cited *C. lallanii*, *FL* is numerically identical to *FI* for every use and would add no information. The relative importance index (*RI*) [42] and the rank order priority (*ROP*) [41] rank species against one another and cannot be calculated meaningfully for one species, and the cultural importance index (*CI*) [37] reduces to *UV* when only one species is studied. Free-list salience indices need the order in which uses were listed, which was not recorded, and similarity indices such as the Jaccard index need comparable use-report data from other communities, which do not yet exist for this species; the cross-cultural comparison (Section 4.5) is therefore qualitative [43].

## 3. Results

### 3.1. Socio-Demographic Characteristics of the Participants

**Figure 2:**
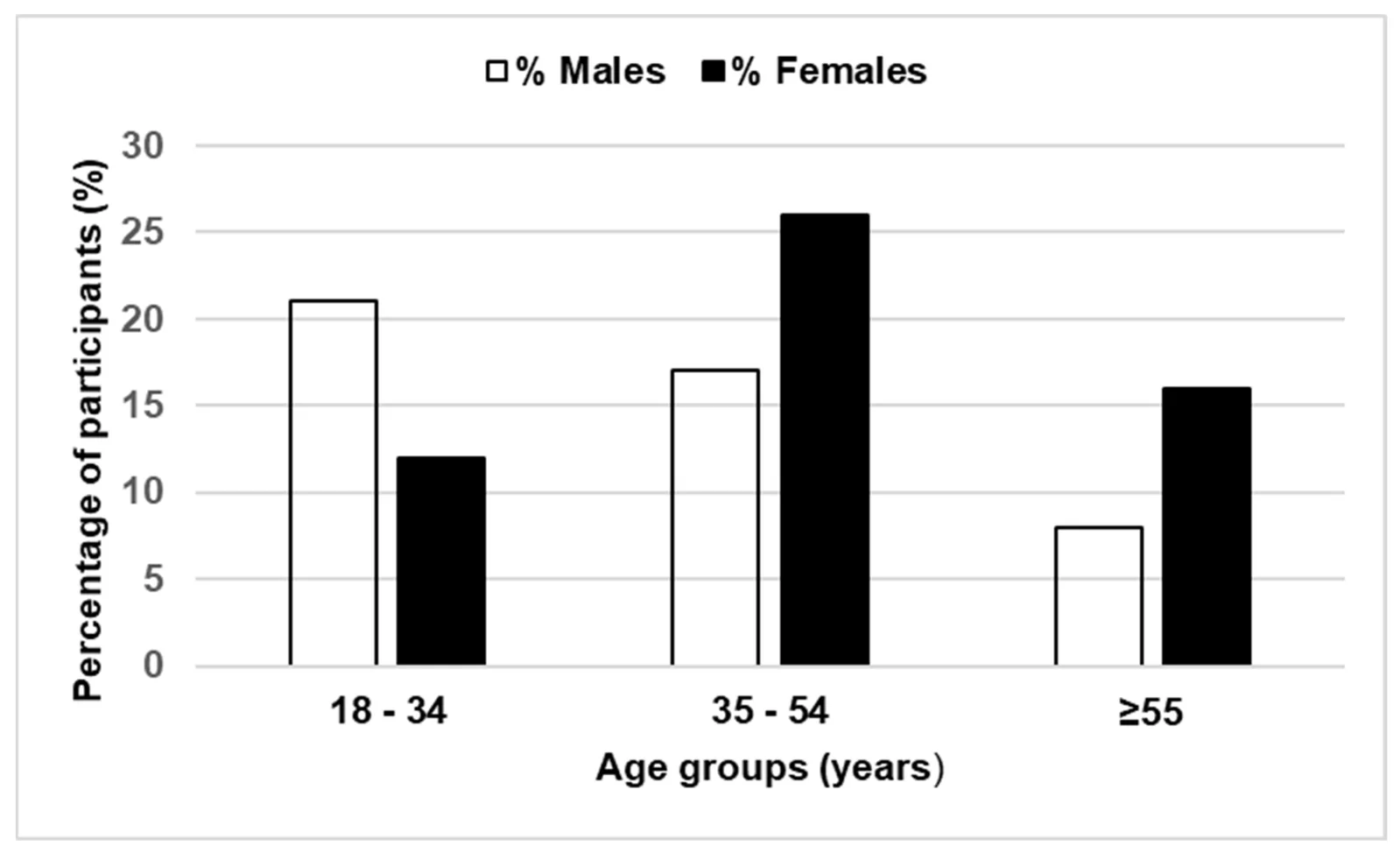
Gender and age of interviewees (N = 100).

A total of 100 participants from Imbizo, Maqeleni, and UMlalazi communities at Eshowe were interviewed to assess their knowledge of the traditional food, medicinal, cultural, and economic uses of *C. lallanii* (Figure 2). The study consisted of more female (54%) than male (46%) participants. More males were interviewed compared with females among the youth, but the opposite pattern was recorded among the middle-aged and elderly participants.

### 3.2. Uses of Canthium lallanii

a. Food uses

**Figure 3:**
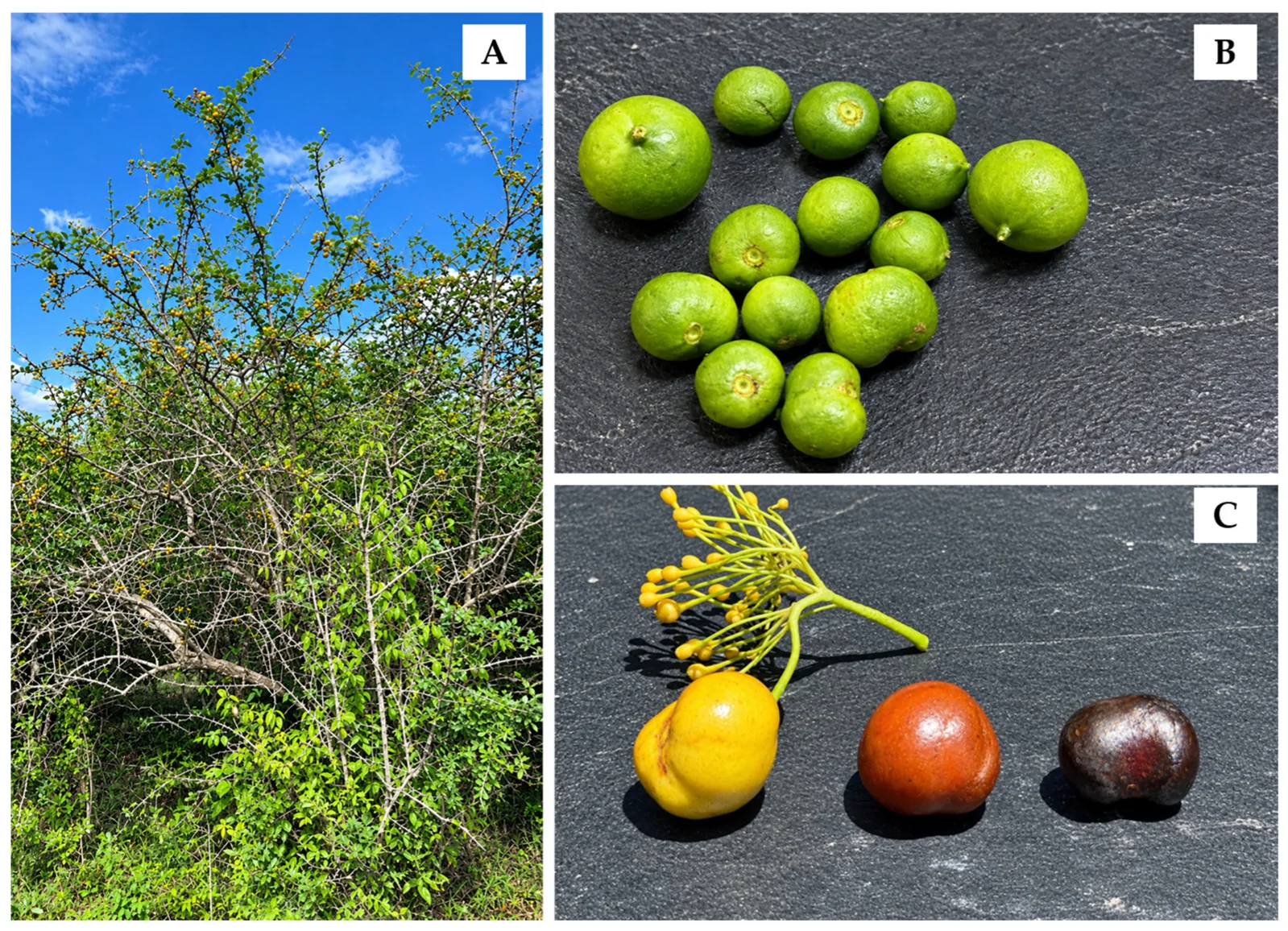
Morphological characteristics of *Canthium lallanii*. (**A**) General growth form of the plant, showing its overall habit and branching pattern; (**B**) unripe fruits, showing their immature developmental stage and green coloration; and (**C**) ripe fruits, showing the mature stage characterized by a change in fruit colour and colour variation in ripe fruits. Photographs: Bahle Ngiphiwe Msomi (first author).

Participants wait for the fruits to change colour from green to yellowish-brown as a sign of ripening (Figure 3) before picking them from the tree for direct consumption. After collection, the fruit is eaten directly, and the seeds are discarded (Table 1). *Canthium lallanii* was recognized and used by the entire sample (*FI* = 100%). Participants mentioned that fruits are usually sold in markets and on roadsides for $ 0.18 in small plastic bags or $ 0.30 per small cup, representing a modest but locally meaningful income stream. The fruit pulp is also used in the preparation of traditional beer, added during the *isijingi* stage of umqombothi (traditional maize-sorghum beer) brewing to enhance the beverage’s flavour. *Canthium lallanii* fruits are incorporated at the stage traditionally used by the community, as crushed pulp. The fruit is first cleaned to remove undesirable or inedible portions, and the seeds are removed before being added to the fermenting beverage. Fermentation is allowed to continue until the desired flavour, aroma, acidity, and alcohol characteristics are achieved. The resulting beverage is not only a fermented cereal product but also a culturally embedded food practice through which flavour is modified and potentially contributes additional sensory and nutritional attributes. Participants described the contribution of the fruit in sensory (flavour) terms; the physicochemical and microbial effects of the pulp on the beverage were not measured in this study and are discussed in Section 4.2.

b. Medicinal uses

Participants stated that they use the roots for body cleansing, by chopping and placing the roots in warm bathing water (Table 1). Roots are boiled to make a decoction used as a mouthwash or crushed and inserted into an affected tooth to relieve toothache. Roots can also be crushed or ground into powder and boiled to make a decoction that treats pimples or acne by steaming. Leaves are boiled with water, and the decoction is sipped to relieve a stomachache. Fresh leaves of *C. lallanii* are infused in hot water to produce a herbal tea traditionally consumed to regulate diabetes, and fruits are also crushed or milled into a fine powder and subsequently boiled to prepare a decoction that is traditionally ingested to control diabetes. This layered medicinal repertoire illustrates how a single plant sustains a broad, self-managed system of household healthcare independent of formal healthcare access.

c. Other uses

Participants stated that dry *C. lallanii* makes an excellent, largely smoke-free fire, though some homesteads reported the wood as unsuitable for firewood without specifying why (Table 1). The branches and stems are also used to construct homesteads, fences, and kraals, and to produce crafts including slingshots, broom handles, knobkerries or fighting sticks, and wooden utensils. These crafted items are sold in markets or on roadsides, further embedding the species within local livelihood strategies.

d. Quantitative ethnobotanical indices

The 100 participants provided a total of 184 use reports across 15 distinct uses, giving an overall use value of 1.84; on average, each participant reported nearly two uses of the species (Table 2). Food uses contributed the most use reports (*UV* = 1.01), followed by material uses (*UV* = 0.77), whereas medicinal uses contributed very few (*UV* = 0.06). *RFC* values (FI/100; Table 1) ranged from 1.00 for the edible fruit and 0.46 for firewood to 0.01–0.02 for each medicinal use. Informant consensus was very high for food uses (*ICF* = 0.99) and high for material uses (*ICF* = 0.91), but low for medicinal uses (*ICF* = 0.20), because each of the five medicinal uses was reported by only one or two participants.

### 3.3. Plant Parts Used

**Figure 4:**
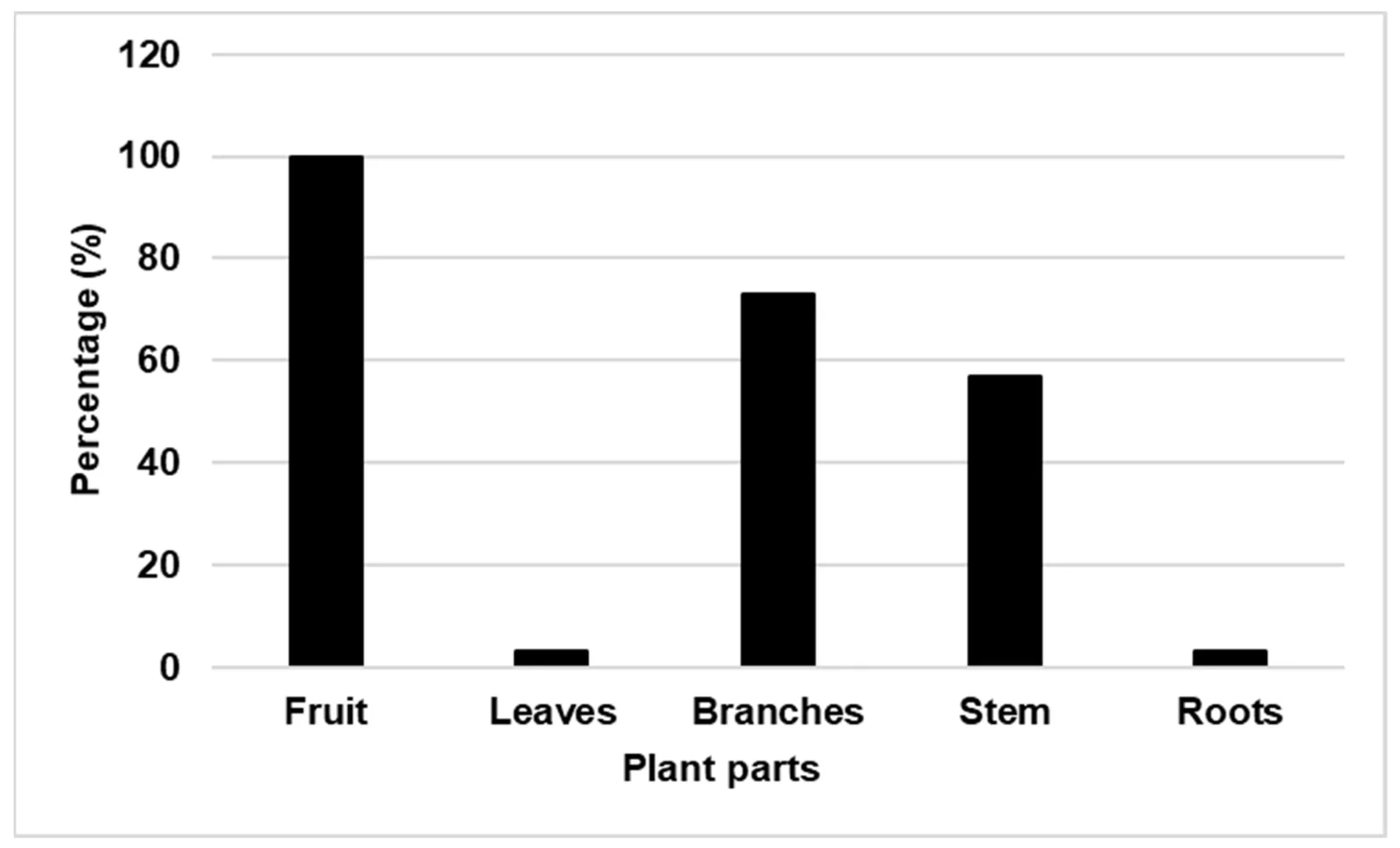
Plant parts of *C. lallanii* used for different purposes.

Five plant parts were used for diverse purposes (Figure 4). The three most frequently used were fruit (100%), branches (73%), and stem (57%), while leaves (3%) and roots (3%) were less commonly drawn upon. Fruits are consumed mainly as a snack and, together with leaves and roots, for medicinal purposes, while branches and stems are used chiefly for firewood and crafts. Participants also mentioned that *C. lallanii* thorns contain a liquid poison that is considered highly dangerous—a further dimension of the practical ecological knowledge held about the species.

### 3.4. Seasonal Availability and Occurrence of Fruits in the Study Area

**Figure 5:**
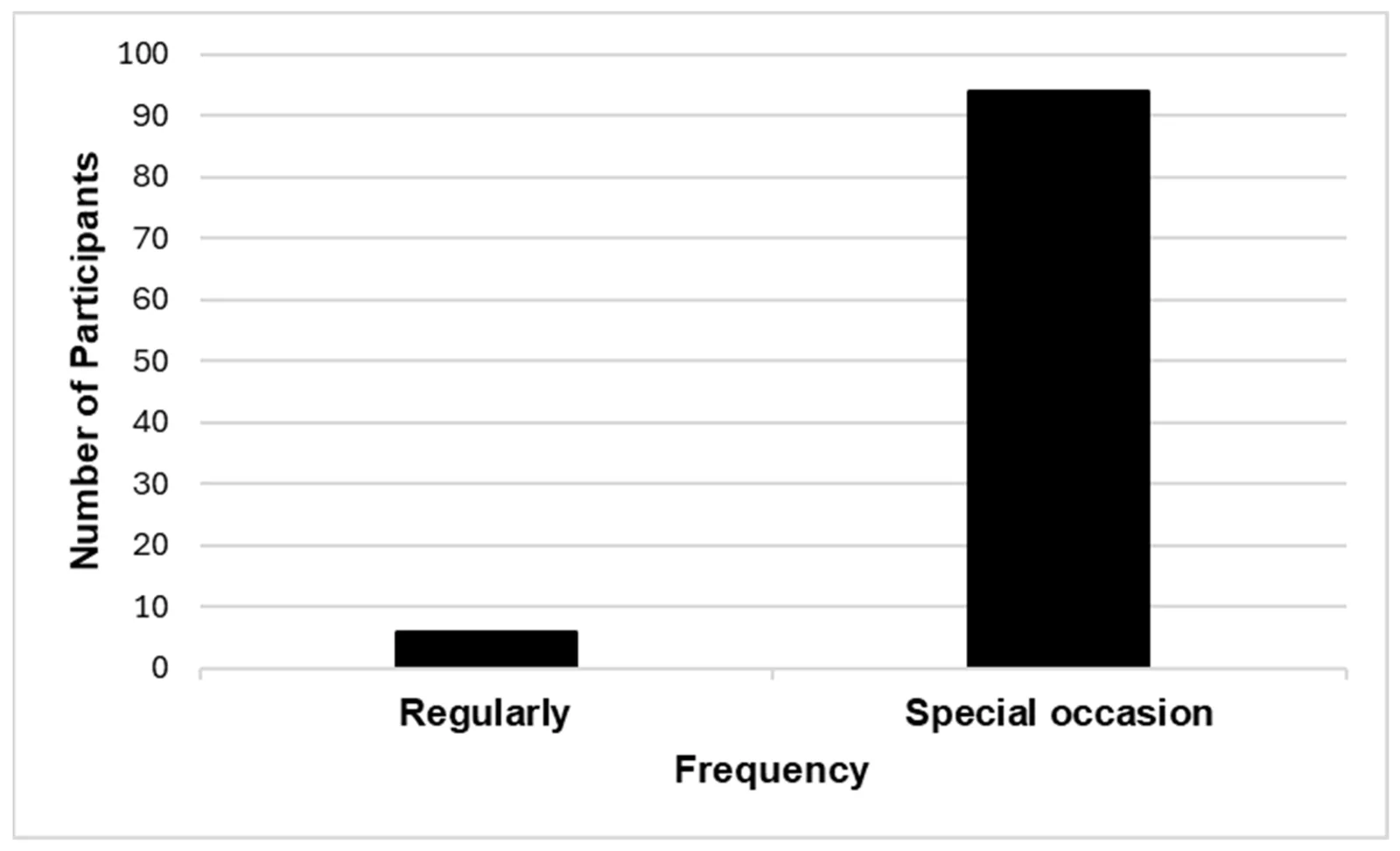
Frequency of *C. lallanii* fruit consumption. Bars show numbers of participants (*N* = 100); because *N* = 100, counts are numerically equal to percentages.

Fruits of *C. lallanii* become available during summer, corresponding to the species’ typical annual ripening period, a pattern that participants described as consistent from year to year. Almost all participants reported that fruits are primarily consumed during the summer season (Figure 5), with interannual variation in yield linked to rainfall, higher precipitation producing greater yields, and limited rainfall reducing production. A few participants indicated that fruits are nonetheless consumed regularly, regardless of annual variation.

**Figure 6:**
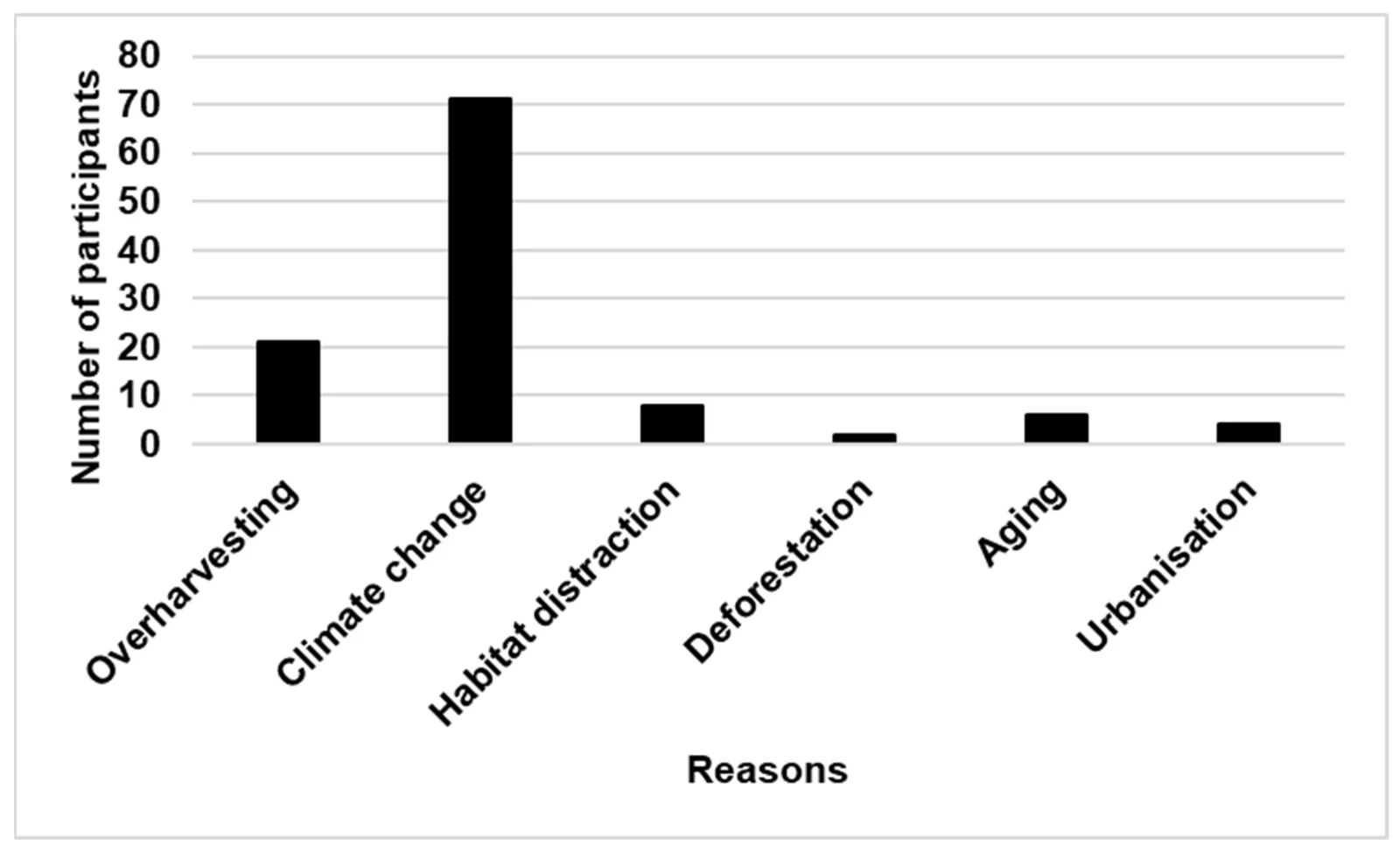
Reasons for changes in *C. lallanii* fruit availability. Bars show numbers of participants (*N* = 100); because *N* = 100, counts are numerically equal to percentages.

Participants identified several converging drivers of change in *C. lallanii* availability over time (Figure 6). Climate change was identified as the principal driver, with participants directly linking rainfall patterns to fruit yield. Overharvesting was also cited as contributing to declining availability. Urbanization was described as disrupting habitat, as some community members clear trees (deforestation) for land development, directly reducing the fruit plant’s presence. Participants reported that this has visibly reduced the number of fruiting plants they observe. The natural ageing of remaining stands, compounded by a lack of active management or replanting, was identified as a further contributing factor.

Despite these pressures, participants articulated concrete, community-generated strategies for conserving and sustaining *C. lallanii*, including limiting urbanization and deforestation, planting the species within homesteads and protected areas, and preventing overharvesting and habitat destruction. Many emphasized the importance of documenting and actively transmitting traditional knowledge, particularly regarding the plant’s nutritional value to younger generations, suggesting that this be done through social media platforms, by encouraging elders to pass on their knowledge directly, and by compiling traditional uses of *C. lallanii* for future reference. Participants also recommended cultivating the species in botanical gardens and nurseries to promote public education and awareness, reflecting a clear community mandate for knowledge preservation alongside biological conservation.

## 4. Discussion

### 4.1. Socio-Demographic Characteristics of the Participants

Socio-demographic patterns are discussed here because the distribution of knowledge by gender and age shows who currently holds, and who is likely to transmit, knowledge of *C. lallanii*. This addresses objective (ii) of the study (by whom the species is used) and directly informs the knowledge-transmission strategies proposed by participants (Section 4.4). The larger proportion of female participants interviewed about *C. lallanii* (Figure 2) is consistent with research on *Strychnos spinosa* [44], where women particularly in rural areas are more actively engaged in the collection and use of indigenous fruit plants. Women hold extensive indigenous knowledge and are skilled in collecting, processing, and managing plants, likely reflecting their participation in agricultural and cultural practices and their roles within traditional food systems and household resource management [45,46]. This gendered distribution of knowledge carries direct implications for who currently safeguards, and who is best positioned to transmit, this traditional food knowledge going forward. The higher proportion of male participants among the youth may reflect that young males engaged in cattle herding spend more time in wild spaces, increasing their exposure to plant resources relative to young females, who are more often engaged in domestic activities [47]. This pattern suggests knowledge transmission pathways may themselves be shifting along generational and gendered lines, with implications for how such knowledge is documented and safeguarded.

### 4.2. Uses of Canthium lallanii

The primary consumption of *C. lallanii* fruits as a snack in the current study is consistent with earlier findings [15,18], reinforcing the species’ longstanding place within local food culture. Its commercialization in local markets and on roadsides (Table 1) reflects a broader practice among local communities of generating income and employment through the sale of indigenous fruits [46]. Across southern Africa, indigenous fruits represent a significant income source, particularly for less privileged households [48,49], and carry real potential to boost rural incomes and employment [49] as either a supplementary or a primary source of household earnings [50]. This highlights how traditional plant knowledge continues to function as a practical resilience strategy rather than a purely historical practice. The use of *C. lallanii* fruit pulp in traditional beer preparation parallels similar uses of the fruit pulp of *Diospyros mespiliformis*, *Grewia flava*, *Parinari capensis*, *Ziziphus mucronata* [51], *Harpephyllum caffrum*, *Carissa bispinosa*, *Phoenix reclinata*, *Opuntia ficus-indica*, *Strychnos spinosa*, *Syzygium cordatum*, *Ximenia caffra*, *Mimusops zeyheri* [49], *Sclerocarya birrea*, *Berchemia discolor*, and *Vangueria infausta* [52] in fermented beverages, situating *C. lallanii* within a wider regional tradition of plant-based fermentation practice that carries strong ritual and communal significance.

From a food science perspective, the use of *C. lallanii* pulp in umqombothi warrants closer attention. Umqombothi is typically brewed from maize meal and sorghum malt in two fermentation phases: a spontaneous lactic acid fermentation (souring) of the mash, followed by cooking into a porridge, cooling, addition of further malt, and an alcoholic fermentation dominated by yeasts, principally *Saccharomyces cerevisiae* [53]. Ripe fruit pulp added at the porridge stage would be expected to supply fermentable sugars that support yeast activity, organic acids that lower pH and reinforce the characteristic sour taste, and volatile and phenolic compounds that modify aroma, colour, and astringency; wild yeasts and lactic acid bacteria carried on fruit surfaces may also enter the fermentation [51,53]. None of these effects has yet been measured for *C. lallanii*. Compositional data for the genus are limited and derive mostly from Asian species: leaves of *C. coromandelicum* (syn. *C. parviflorum*) are rich in polyphenols, flavonoids, β-carotene, α-tocopherol, and ascorbic acid [54]; methanolic extracts of its fruits show in vitro antioxidant activity [55]; the fruit volatiles of the West African *C. subcordatum* have been characterized by GC–MS [56]; and the genus as a whole contains flavonoids, terpenoids, and other phenolic compounds [54]. To our knowledge, no proximate, sugar, organic acid, mineral, or phenolic profile has been published for *C. lallanii* fruit, although its palatability [17] and universal consumption at Eshowe indicate clear food potential. Characterizing these parameters, and the effect of the pulp on the pH, sugar utilization, microbial succession, and sensory profile of umqombothi, is a priority for future work (Section 4.6). The use of *C. lallanii* roots for general body cleansing (Table 1) mirrors comparable practice with the roots, bark, and leaves of *Strychnos gerrardii* among the Emkhwandlwini community [34]. Current findings support earlier documentation of *C. lallanii* leaves used to treat stomachache [15,17] and leaf infusion used against diarrhea [57]. The treatment of pimples or acne with *C. lallanii* roots at Eshowe parallels the use of *Averrhoa bilimbi* fruit for the same purpose in South Aceh, Indonesia [22], pointing to convergent, independently developed plant-based health knowledge across geographically distant traditional food systems. The use of *C. lallanii* leaves or fruits to regulate diabetes at Eshowe is comparable to the use of *S. birrea* fruits for the same purpose in the Ohangwena and Oshikoto regions of Northern Namibia [26], and the use of *C. lallanii* roots for toothache aligns with documented use of *Ancylanthos rubiginosus*, *Diospyros lycioides*, *S. birrea*, and *Anacardium occidentale* for the same purpose [22,26]. The genus *Canthium* is recognized for antioxidant, antimicrobial, anti-diabetic, and hepatoprotective properties [56], and has long been used across African and Asian herbal medicine traditions to treat ailments including diarrhea, leucorrhoea, constipation, snakebite, diabetes, hypertension, venereal disease, and scabies [58]. Collectively, this medicinal repertoire illustrates how traditional plant knowledge continues to provide accessible, self-managed primary healthcare in contexts where formal healthcare access may be limited or costly.

The medicinal uses recorded here should nonetheless be interpreted with caution. Each medicinal application was reported by only one or two of the 100 participants (FI = 1–2%; Table 1), and the medicinal category showed low informant consensus (ICF = 0.20; Table 2), in contrast to the near-complete consensus for food (ICF = 0.99) and material uses (ICF = 0.91). Because use data were obtained by free recall rather than from a prompted checklist, these low values do not result from participants being asked about uses they did not know; rather, they indicate that medicinal knowledge of *C. lallanii* is idiosyncratic or held by a small number of individuals rather than widely shared. Free recall can also under-report less salient uses, so the true extent of this knowledge may be somewhat greater than the FI values suggest. Single-informant reports cannot be regarded as community consensus, but they merit documentation precisely because rarely held knowledge is the most vulnerable to loss, and use against stomach complaints is corroborated by independent earlier records [15,57]. Pharmacological validation, particularly of the anti-diabetic claim, is required before any therapeutic value can be inferred.

The use of *C. lallanii* branches and stems for firewood (Table 1) reflects the continued reliance of rural households and small-scale food industries on firewood as an energy source [22,59], a pattern also documented in South Aceh and in three villages of Mpumalanga Province, South Africa [22,50]. Even where electricity is available, some households continue to rely on fire for cooking and heating water for bathing [46], suggesting that traditional resource use persists as a resilience strategy rather than simply a default in the absence of alternatives. The use of *C. lallanii* stems and branches in construction and craft production aligns with findings from Mpumalanga Province, where indigenous fruit species similarly serve as sources of fodder, firewood, craft materials, and cash income [52]. Indigenous fruit plants more broadly play an important role in supplying construction materials to local livelihoods [60,61], and comparable craft-material uses of indigenous fruit plants have been documented in South Aceh, Indonesia [22]. This is evidence of a shared, cross-regional logic by which traditional food plants sustain not only nutrition but also shelter, tools, and material culture.

### 4.3. Plant Parts Used

The predominance of fruit as the most frequently used plant part (Figure 4) is consistent with other studies in which fruit was similarly the most commonly used part [46,50], and with broader findings that fruits and leaves are the most important plant parts used as food across cultural groups, both in southern Africa and globally [20]. In the Mpumalanga villages study, root use was more strongly linked to therapeutic purposes among communities [50], while across the wider *Canthium* literature, leaves are the plant part most frequently drawn upon in traditional preparations [56]. Prior research similarly documents the use of branches and stems of indigenous fruit plants for firewood, construction, and furniture [22]. This reinforces that the pattern of use recorded at Eshowe sits within a well-established regional template for how traditional communities draw multiple forms of value from a single fruit species.

### 4.4. Seasonal Availability and Occurrence of Fruits in the Study Area

*Canthium lallanii* fruits were reported to be consumed mainly during summer (Figure 5), consistent with findings from Mpumalanga Province that most indigenous fruit species become available during the hot–dry summer season, a period often associated with broader food scarcity [50]. The seasonal availability of indigenous fruits at precisely this point in the year therefore plays a meaningful role in supplementing household diets. This reinforces food security during a vulnerable period, illustrating the practical resilience function that traditional food plants continue to serve. Fruiting seasons and consumption patterns of indigenous fruit plants vary by species [50,62]. Comparable seasonal patterning has been documented elsewhere in Africa, such as in Tigray, Ethiopia, where fruiting of indigenous species in lowland areas typically occurs between October and January, while in midland and highland zones it extends from December to June [63]. This highlights how climatic and ecological gradients shape the rhythms around which traditional food knowledge is organized.

The threats to *C. lallanii* availability identified by participants (Figure 6) mirror well-documented, converging pressures on indigenous fruit species across South Africa, where overharvesting and urbanization are recognized as major drivers of population decline [52,64]. Urbanization drives the construction of houses, roads, industry, and commercial space, frequently through land clearing that destroys natural habitat; these land-use changes contribute to deforestation and the ultimate loss of significant indigenous fruit trees [46], rendering such species vulnerable to overexploitation and genetic impoverishment [65]. Comparable dynamics have been confirmed in Gauteng Province, where urbanization-driven deforestation has negatively affected *Mimusops zeyheri* populations [46], while fruiting phenology and fruit quality more broadly are recognized as highly vulnerable to climate change [66]. This is an emerging threat to food and nutritional security worldwide [67]. Taken together, these pressures illustrate how the erosion of a single indigenous plant species is entangled with wider socio-environmental forces, habitat loss, displacement of land use, and cultural change.

Importantly, however, the Eshowe communities did not present as passive recipients of these pressures. Participants articulated concrete, locally devised conservation strategies that echo practices documented in the Ehlanzeni District of Mpumalanga Province, where communities conserve plants through seed storage, cultivation in home gardens, and harvesting restricted to immediate need, reflecting active stewardship of shared plant resources [52,68]. Domestication of indigenous fruit trees offers one viable pathway to reduce pressure on wild populations [65], and the deliberate integration of promising indigenous fruits into existing land-use systems can meaningfully strengthen rural livelihoods over time [69]. These community-generated responses are best understood not merely as biodiversity conservation measures. Instead, they are understood as expressions of food sovereignty in practice. Local communities assert control over the plant resources, knowledge systems, and food practices most relevant to their own nutritional, economic, and cultural continuity, rather than depending on external actors to define what should be conserved and how [7]. This aligns with the wider argument that food sovereignty, particularly in postcolonial and rural African contexts, functions as a continuation of communities’ efforts to retain self-determination over land and plant-based livelihoods in the face of external pressure [8]. Viewed this way, participants’ calls to document elders’ knowledge, cultivate the species at home, and share information through social media are not incidental suggestions but a coherent, if informal, community strategy for reasserting control over a resource under threat.

Realizing these community aspirations nonetheless requires supportive structures beyond individual household effort. Protecting natural habitats and regulating harvesting activities remain essential to prevent further overexploitation and habitat degradation. Furthermore, the establishment of protected areas or reserves dedicated to indigenous fruit trees can meaningfully safeguard these resources [63]. Partnerships between government agencies, research institutions, non-governmental organizations, and local communities can support knowledge sharing, resource mobilization, and the co-development of sustainable management strategies that keep local communities as the central authority over their own traditional knowledge [63]. Embedding such partnerships within educational programming and public engagement, as participants themselves proposed through social media transmission and elder-led teaching, offers a concrete route by which ethnobotanical documentation can inform alternative food futures, policies, and educational practices.

### 4.5. Cross-Cultural Comparison of Uses

To place the Eshowe findings in a wider cultural context, the uses recorded here were compared with published records of *C. lallanii* from other cultural groups within its range, records for related *Canthium* species used by other cultures in Africa and Asia, and the broader pattern of indigenous fruit use among other cultural groups (Table 3). Because no other quantitative study of this species exists, the comparison is qualitative and based on whether each use appears in the published record.

Within the South African range of the species, which extends from the Eastern Cape through the former Transkei into KwaZulu-Natal [17], two uses are recorded consistently across cultural groups and sources; eating the fresh fruit, and using the leaves against gastrointestinal complaints. Fruit consumption and a leaf infusion for stomach problems and diarrhea have been documented in KwaZulu-Natal and among Swati communities in Eswatini [15], and the plant is used for stomach complaints, dysentery, and diarrhea in Zulu traditional medicine [17,19]. At Eshowe, every participant ate the fruit and leaves were used against stomachache, so these two uses form a shared, cross-cultural core of knowledge about the species. Because the same leaf remedy has been recorded independently in several communities, it is a stronger candidate for pharmacological testing than its low FI at Eshowe alone would suggest. In contrast, adding the fruit pulp to umqombothi, the root remedies for body cleansing, acne, and toothache, the use of leaves and fruit for diabetes, and all eight material uses have not, to our knowledge, been recorded before for this species. These uses may be specific to the Eshowe communities, or they may simply not have been documented elsewhere. The species also has isiXhosa names, including ubuchopho benja and isiphambatho [17], which shows that it is recognized in Xhosa-speaking communities of the Eastern Cape, yet no record of how these communities use it was found. This is a clear gap for future comparative work. A similar pattern also appears for closely related species (*C. inerme*) used by other cultures. *Canthium inerme*, is a southern African species with vernacular names in isiXhosa, isiZulu, Sepedi, and Tshivenda. It also has edible fruit, its leaves are used for stomach ailments, and its hard, heavy wood has been used for furniture and wagon-making and as shelter for livestock [70].

For indigenous fruits more generally, the Eshowe profile, in which fresh fruit is eaten as a snack and the same plant provides secondary medicinal, fuel, and craft uses, recurs in studies among other Zulu-speaking communities for *Strychnos spinosa* [44] and *S. gerrardii* [34], among Mapulana communities in Mpumalanga [35], Setswana-speaking communities in North West Province [13], and Owambo communities in northern Namibia [26], and outside southern Africa in Ethiopia [22,63] and Indonesia [22]. Using fruit to flavour or ferment beverages is likewise shared by many southern African cultural groups and involves a wide range of species [20,51,52]; adding *C. lallanii* pulp to umqombothi is a local, previously unrecorded example of this shared tradition. Where Eshowe differs is in the weight given to material uses: most participants used the branches and stems, mainly for firewood, whereas medicinal knowledge was rare. At Eshowe, therefore, the species is valued as much as a household material resource as it is as a food, which may reflect local demand for fuel and building material as well as cultural preference.

The comparison distinguishes two layers of knowledge about *C. lallanii*: a widely shared core (edible fruit and leaf remedies for the gut) that is recognized across cultural groups and mirrored in related species elsewhere, and a set of uses (beer flavouring, root remedies, and most material uses) documented so far only at Eshowe. The shared core shows where knowledge is most robust; the locally specific uses are the most vulnerable to loss, because no independent record of them exists. Quantitative surveys using the same indices among Xhosa-speaking communities in the Eastern Cape and among Zulu communities elsewhere in KwaZulu-Natal would allow use similarity (for example, the Jaccard index) and consensus values to be compared directly.

### 4.6. Limitations

This study is based on a single geographic cluster of three neighbouring communities within Eshowe, and the frequency indices reported here reflect the perceptions and practices of the 100 participating homesteads rather than the full range of use that may exist across *C. lallanii’s* broader distribution in Southern and East Africa. As with most ethnobotanical surveys relying on self-reported use, recall and social-desirability effects cannot be entirely excluded, and quantitative dietary or phytochemical validation of the reported medicinal applications was beyond the scope of this study. The cross-cultural comparison (Section 4.5) is qualitative, because no comparable quantitative data exist for other communities, and the absence of a use from the published record does not show that the use is absent in practice. Use data were obtained by free recall, which can under-report less salient uses, so the low frequency indices recorded for medicinal uses should be read as minimum estimates of how widely this knowledge is held. In addition, no compositional analysis of the fruit or of fruit-amended beer was undertaken. Future research could usefully pair this ethnobotanical documentation with proximate, sugar, organic acid, mineral, and phenolic analyses of the fruit and with controlled brewing trials quantifying the effect of the pulp on the pH, sugar utilization, microbial succession, and sensory profile of umqombothi, with phytochemical and pharmacological validation of the reported medicinal uses, with population and regeneration surveys to quantify the species’ conservation status more precisely, and with comparative ethnobotanical work across other communities where *C. lallanii* occurs, to establish how consistent these use patterns and threats are at a regional scale. Participatory approaches that directly involve elders and youth in co-designing knowledge-transmission tools (for example, community-led digital or oral-history archives) would also extend the conservation strategies that participants themselves proposed.

## 5. Conclusions

*Canthium lallanii* is an indigenous fruit plant whose food, medicinal, and material uses continue to sustain household nutrition, income, and cultural practice among Eshowe communities of KwaZulu-Natal. Its core uses, the edible fruit and leaf remedies for gastrointestinal complaints, are shared with other cultural groups and mirrored in related *Canthium* species elsewhere, whereas beer flavouring, root remedies, and most material uses appear specific to the Eshowe communities and are therefore the knowledge most at risk of loss. Beyond its immediate practical value, the species anchors a body of traditional food knowledge that reflects and reproduces community identity, self-reliance, and intergenerational continuity. This is precisely the kind of localized resilience strategy through which communities sustain autonomy and dignity amid broader socio-environmental pressure. Yet this study shows that *C. lallanii’s* availability has visibly declined due to the combined effects of urbanization, overharvesting, climate change, habitat destruction, ageing plant populations, and deforestation, placing both the species and the knowledge attached to it at real risk of loss. Community members have already begun articulating their own responses. These responses include domestication, homestead cultivation, and active transmission of knowledge to younger generations that constitute a grounded, community-driven form of food sovereignty in action. Supporting and scaling these locally generated strategies, alongside documenting and cultivating the species in botanical gardens for public education, offers a concrete and achievable pathway for safeguarding both *C. lallanii* and the traditional ecological knowledge that surrounds it.

At policy level and through practice, three implications follow directly from these findings. First, local government and conservation agencies in the uMlalazi Municipality could support homestead and community-level propagation of *C. lallanii*, rather than relying solely on wild harvesting. This can be done by supplying seedlings or propagation training through existing agricultural extension structures. Second, schools and community organizations in Eshowe could formalize the knowledge-transmission channels proposed by participants, elder-led teaching sessions and simple digital or oral-history recording to prevent the loss of food, medicinal and craft knowledge as older knowledge-holders age. Third, given that similar pressures and community responses are documented for other southern African indigenous fruit species, regional conservation and food-security policy would benefit from treating underutilized indigenous fruits as a category meriting coordinated support, rather than addressing each species in isolation. Together, these steps would contribute to broader efforts to recognize and activate traditional food knowledge as a pillar of sustainable, community-controlled, and just food systems.

## Figures and Tables

**Table 1:** Ethnobotanical uses of *C. lallanii* (N = 100).

Category	Uses	Plant Part Used	Method of Preparation	Administration	*FI* (%)
Food	Edible fruit	Fruit	Ripe fruit is directly eaten, and seeds are discarded	Orally	100
	Isijingi	Fruit pulp	Mixed with traditional beer	Orally	1
Medicine	Regulates diabetes	Leaves/fruits	Fresh application, crushed/ground into powder then boiled (decoction)	Tea	2
	Cleansing	Roots	Soaked in water	Bathing	1
	Pimples/acne	Roots	Crushed/ground into powder then boiled (decoction)	Steaming	1
	Stomachache	Leaves	Boiled (decoction)	Orally	1
	Toothache	Roots	Boil/crush the roots and grind them into powder, then insert into the tooth	Mouthwash	1
Other	Firewood	Branches/stem	Burn dry branches or stems to make fire	Not Applicable	46
	Knobkerries/fighting sticks	Branches	Branches cut, shaped, and dried into knobkerries and fighting sticks	Not Applicable	8
	Homestead	Branches/stem	Branches/stems cut and used as poles and frames in homestead construction	Not Applicable	6
	Kraals	Branches/stem	Branches/stems cut and erected as kraal poles and barriers	Not Applicable	5
	Wooden utensils	Branches	Branches carved into wooden utensils	Not Applicable	5
	Fencing	Branches	Spiny branches cut and used as fence posts or barriers	Not Applicable	4
	Slingshots	Branches	Forked branches cut and shaped into slingshot frames	Not Applicable	2
	Broom handle	Branches	Straight branches cut and smoothed into broom handles	Not Applicable	1

*FI*, frequency index Equation (1); *RFC* = *FI*/100. “Not applicable” denotes non-consumptive (material) uses, for which no route of administration applies.

**Table 2:** Use reports, use value (*UV*), and informant consensus factor (*ICF*) for each use category of *C. lallanii* (*N* = 100).

Use Category	Use Reports (*Nur*)	Distinct Uses (*Nt*)	*ICF*	*UV*
Food	101	2	0.99	1.01
Medicine	6	5	0.20	0.06
Other (material)	77	8	0.91	0.77
All uses	184	15	—	1.84

*Nur*, number of use reports (sum of *FC* values in the category); *Nt*, number of distinct uses in the category; *ICF* = (*Nur − Nt*)/(*Nur −* 1); *UV* = *Nur/N* with *N* = 100.

**Table 3:** Cross-cultural comparison of the uses recorded for *C. lallanii* at Eshowe with records from other cultural groups and for the related *C. inerme*.

Taxon	Cultural Group	Language	Region	Food Uses	Medicinal Uses	Material and Other Uses	Ref.
*C. lallanii*	Zulu	IsiZulu	Eshowe, KwaZulu-Natal	Fresh fruit eaten as a snack; pulp added to umqombothi	Roots: body cleansing, acne, toothache; leaves: stomachache; leaves and fruit: diabetes	Firewood, homestead poles, kraals, fencing, knobkerries, utensils, slingshots, broom handles	This study
*C. lallanii*	Zulu and Swati	IsiZulu and IsiSwati	KwaZulu-Natal and Eswatini	Fresh fruit	Leaf infusion for stomach problems and diarrhea	Not recorded	[15]
*C. lallanii*	Zulu and Xhosa	IsiZulu and IsiXhosa	KwaZulu-Natal and Eastern Cape	Fresh fruit (taste likened to dried peach)	Stomach complaints, dysentery, diarrhea	Hedging; heavy, fine-grained wood	[17,19]
*C. lallanii*	Xhosa	IsiXhosa	Eastern Cape	Not recorded	Not recorded	Not recorded	[17]
*C. inerme*	Xhosa; Zulu; Pedi and Venda	IsiXhosa; Isizulu; Sepedi and Tshivenda	Eastern Cape; KwaZulu-Natal and Limpopo	Fresh fruit	Leaves for stomach ailments	Furniture and wagon-making; shelter for livestock	[70]

“Not recorded”: no use in this category was found in the cited source(s); “Not specified”: the source does not separate uses by category.

## Data Availability

The original contributions presented in this study are included in the article. The raw interview data contain information about individual participants and are not publicly available in order to protect participant privacy and confidentiality. De-identified data are available from the corresponding author upon reasonable request.

## Abbreviations

The following abbreviations are used in this manuscript:

FI	Frequency Index
FC	Frequency of citation
RFC	Relative frequency of citation
UV	Use value
ICF	Informant consensus factor
Nur	Number of use reports
Nt	Number of distinct uses
°S	Degrees South
°E	Degrees East
mm	Millimetre
°C	Degrees Celsius
%	Percentage

## References

[1] Oniang’o R., Allotey J., Malaba S.J. (2004). Contribution of indigenous knowledge and practices in food technology to the attainment of food security in Africa. J. Food Sci..

[2] Mothupi F.M., Shackleton C.M. (2025). Traditional knowledge and consumption of wild edible plants in rural households, Limpopo Province, South Africa. J. Ethnobiol. Ethnomed..

[3] Nathani-Wane N. (2014). Indigenous African Knowledge Production: Food-Processing Practices Among Kenyan Rural Women.

[4] Shashikant M.B.S. (2025). Agricultural Transformation for Sustainable Development in India: Challenges, Innovations, and Policy Imperatives. IIP Int. Multidiscip. Res. J..

[5] Reyes-García V., Guèze M., Luz A.C., Paneque-Gálvez J., Macía M.J., Orta-Martínez M., Pino J., Rubio-Campillo X. (2013). Evidence of traditional knowledge loss among a contemporary indigenous society. Evol. Hum. Behav..

[6] Aswani S., Lemahieu A., Sauer W.H.H. (2018). Global trends of local ecological knowledge and future implications. PLoS ONE.

[7] Wittman H. (2011). Food sovereignty: A new rights framework for food and nature?. Environ. Soc. Adv. Res..

[8] Grey S., Patel R. (2015). Food sovereignty as decolonization: Some contributions from Indigenous movements to food system and development politics. Agric. Hum. Values.

[9] McKinley C.E., Walters K.L. (2023). “It’s always about sharing, and caring, and loving, and giving”: Decolonized and transcendent indigenist foodways fostering health and resilience. Advers. Resil. Sci..

[10] Addis G., Asfaw Z., Woldu Z. (2013). The Role of Wild and Semi-wild Edible Plants to Household Food Sovereignty in Hamar and Konso Communities, South Ethiopia. Ethnobot. Res. Appl..

[11] Gonçalves M., Hanazaki N. (2023). Afro-diasporic ethnobotany: Food plants and food sovereignty of Quilombos in Brazil. Ethnobot. Res. Appl..

[12] Awoke A., Dessie Y., Tilahun A., Getachew T. (2026). Ethnobotanical study of wild and semi-wild edible plants and their contributions to food security in Zigem District, Ethiopia. J. Ethnobiol. Ethnomed..

[13] Nko K.I., Sebogo N., Mwinga J.L., Asong J.A., Struwig M., Amoo S.O., Aremu A.O. (2026). Wild edible plants and associated indigenous knowledge among the Setswana-speaking people of North West Province in South Africa. J. Ethnobiol. Ethnomed..

[14] Boon R. (2010). Pooley’s Trees of Eastern South Africa a Complete Guide. Flora and Fauna Publication Trust, South Africa.

[15] Corrigan B.M., Van Wyk B.E., Geldenhuys C.J., Jardine J.M. (2011). Ethnobotanical plant uses in the KwaNibela Peninsula, St Lucia, South Africa. S. Afr. J. Bot..

[16] Singh R.K. (2024). Nomenclatural novelties in Rubiaceae of Old World. J. Biodivers. Conserv..

[17] South African National Biodiversity Institute (SANBI) *Canthium spinosum* PlantZAfrica. https://pza.sanbi.org/canthium-spinosum.

[18] Okem A. (2011). Pharmacological Activities of Selected South African Medicinal Plants. Master’s Thesis.

[19] Hutchings A., Scott A.H., Lewis G., Cunningham A.B. (1996). Zulu Medicinal Plants: An Inventory.

[20] Welcome A.K., Van Wyk B.E. (2019). An inventory and analysis of the food plants of southern Africa. S. Afr. J. Bot..

[21] Rahman I.U., Afzal A., Iqbal Z., Ijaz F., Ali N., Shah M., Ullah S., Bussmann R.W. (2019). Historical perspectives of ethnobotany. Clin. Dermatol..

[22] Suwardi A.B., Navia Z.I., Harmawan T., Syamsuardi, Mukhtar E. (2020). Ethnobotany and conservation of indigenous edible fruit plants in South Aceh, Indonesia. Biodivers. J. Biol. Divers..

[23] Ijaz F., Iqbala Z., Rahmana I.U., Alama J., Khanb S.M., Shaha G.M., Khana K., Afzal A. (2016). Investigation of traditional medicinal floral knowledge of Sarban Hills, Abbottabad, KP, Pakistan. J. Ethnopharmacol..

[24] Tabuti J.R.S., Kukunda C.B., Kaweesi D., Kasilo O.M.J. (2012). Herbal medicine use in the districts of Nakapiripirit, Pallisa, Kanungu, and Mukono in Uganda. J. Ethnobiol. Ethnomed..

[25] Dejene T., Agamy M.S., Agúndez D., Martin-Pinto P. (2020). Ethnobotanical survey of wild edible fruit tree species in Lowland Areas of Ethiopia. Forests.

[26] Cheikhyoussef A., Embashu W. (2013). Ethnobotanical knowledge on indigenous fruits in Ohangwena and Oshikoto regions in Northern Namibia. J. Ethnobiol. Ethnomed..

[27] Gumede T.K. (2019). Heritage tourism as a strategy for the local economic development in KwaZulu-Natal, South Africa. Afr. J. Hosp..

[28] Mbobo T., Richardson D.M., Gwynne-Evans D., Deacon J., Wilson J.R.U. (2022). *Psidium cattleyanum* (Myrtaceae) invasions in South Africa: Status and prognosis. S. Afr. J. Bot..

[29] uMlalazi Municipality IDP (2020). Development of a Nodal Plan for Eshowe: UMlalazi Local Municipality.

[30] Dlamini N. (2021). Land Use Effects on Characteristics and Mineralisation of Nitrogen and Phosphorus During Incubation of Humic Topsoil from Selected Sites in KwaZulu-Natal, South Africa. Master’s Thesis.

[31] Statistics South Africa (2024). Census 2022 Provincial Profile: KwaZulu-Natal.

[32] Statistics South Africa UMlalazi Local Municipality: Census 2011 Key Statistics. https://www.statssa.gov.za/?page_id=993&id=umlalazi-municipality.

[33] Makwana D., Engineer P., Dabhi A., Chudasama H. (2023). Sampling methods in research: A review. Int. J. Trend Sci. Res. Dev..

[34] Mpungose A.C., Zharare G.E., Zimudzi C., Ntuli N.R. (2024). Indigenous knowledge on the uses and morphological variation among *Strychnos gerrardii* N.E.Br. morphotypes at Emkhandlwini area, KwaZulu-Natal, South Africa. Ethnobot. Res. Appl..

[35] Mashile S.P., Tshisikhawe M.P., Masevhe N.A. (2019). Indigenous fruit plants species of the Mapulana of Ehlanzeni district in Mpumalanga province, South Africa. S. Afr. J. Bot..

[36] Masekoameng M.R., Molotja M.C. (2019). The role of indigenous foods and indigenous knowledge systems for rural households’ food security in Sekhukhune District, Limpopo province, South Africa. J. Consum. Sci..

[37] Tardío J., Pardo-de-Santayana M. (2008). Cultural importance indices: A comparative analysis based on the useful wild plants of Southern Cantabria (Northern Spain). Econ. Bot..

[38] Phillips O., Gentry A.H., Reynel C., Wilkin P., Gálvez-Durand C. (1994). Quantitative ethnobotany and Amazonian conservation. Conserv. Biol..

[39] Trotter R.T., Logan M.H., Etkin N.L. (1986). Informant consensus: A new approach for identifying potentially effective medicinal plants. Plants in Indigenous Medicine and Diet: Biobehavioral Approaches.

[40] Heinrich M., Ankli A., Frei B., Weimann C., Sticher O. (1998). Medicinal plants in Mexico: Healers’ consensus and cultural importance. Soc. Sci. Med..

[41] Friedman J., Yaniv Z., Dafni A., Palewitch D. (1986). A preliminary classification of the healing potential of medicinal plants, based on a rational analysis of an ethnopharmacological field survey among Bedouins in the Negev Desert, Israel. J. Ethnopharmacol..

[42] Bennett B.C., Prance G.T. (2000). Introduced plants in the indigenous pharmacopoeia of northern South America. Econ. Bot..

[43] Hoffman B., Gallaher T. (2007). Importance indices in ethnobotany. Ethnobot. Res. Appl..

[44] Mbhele Z., Zharare G.E., Zimudzi C., Ntuli N.R. (2022). Indigenous knowledge on the uses and morphological variation among *Strychnos spinosa* Lam. at Oyemeni Area, KwaZulu-Natal, South Africa. Sustainability.

[45] Kainer K.A., Duryea M.L. (1992). Tapping women’s knowledge: Plant resource use in extractive reserves, Acre, Brazil. Econ. Bot..

[46] Matlala M.E., Ndhlovu P.T., Mokgehle S.N., Otang-Mbeng W. (2024). Ethnobotanical investigation of *Mimusops zeyheri*, an underutilized indigenous fruit tree in Gauteng Province, South Africa. Sustainability.

[47] Uprety Y., Poudel R.C., Shrestha K.K., Rajbhandary S., Tiwari N.N., Shrestha U.B., Asselin H. (2012). Diversity of use and local knowledge of wild edible plant resources in Nepal. J. Ethnobiol. Ethnomed..

[48] Mithöfer R.D., Kenny O., Smyth T.J., Milella L., Hossain M.B., Diop M.S., Rai D.K., Brunton N.P. (2013). Profiling of phytochemicals in tissues from *Sclerocarya birrea* by HPLC-MS and their link with antioxidant activity. ISRN Chromatogr..

[49] Mutelo C., Nyau V., Choobe B., Marinda P., Hachibamba T. (2023). Indigenous knowledge on utilisation aspects of selected edible wild fruits from Zambia. J. Food Res..

[50] Shai K.N., Ncama K., Ndhlovu P.T., Struwing M., Aremu A.O. (2020). An exploratory study on the diverse uses and benefits of locally-sourced fruit species in three villages of Mpumalanga province, South Africa. Foods.

[51] Van Wyk B.E. (2011). The potential of South African plants in the development of new food and beverage products. S. Afr. J. Bot..

[52] Chauke S., Shelembe B.G., Otang-Mbenga W., Ndhlovu P.T. (2024). Ethnobotanical appraisal of wild fruit species used in Mpumalanga Province, South Africa: A systematic review. S. Afr. J. Bot..

[53] Hlangwani E., Adebiyi J.A., Doorsamy W., Adebo O.A. (2020). Processing, characteristics and composition of umqombothi (a South African traditional beer). Processes.

[54] Palvai V.R., Mahalingu S., Urooj A. (2014). *Canthium parviflorum* leaves: Antioxidant activity in food and biological systems, pH, and temperature stability. Chin. J. Biol..

[55] Loganayaki N., Manian S. (2010). In vitro antioxidant properties of indigenous underutilized fruits. Food Sci. Biotechnol..

[56] Tchamgoue J., Tchokokam Y.R.W., Ngouonpe A.W., Ngandjui Y.A.T., Tiani G.L.M., Msagati T.A.M., Ngadjui B.T., Green I.R., Kouam S.F. (2024). The genus *Canthium*: A comprehensive summary on its traditional use, phytochemistry, and pharmacological activities. Fitoterapia.

[57] Okem A., Finnie J.F., Van Staden J. (2012). Pharmacological, genotoxic and phytochemical properties of selected South African medicinal plants used in treating stomach-related ailments. J. Ethnopharmacol..

[58] Essien E.E., Newby J.S., Walker T.M., Setzer W.N., Ekundayo O. (2016). Characterization and antimicrobial activity of volatile constituents from fresh fruits of *Alchornea cordifolia* and *Canthium subcordatum*. Medicines.

[59] Munawaroh E., Saparita R., Purwanto Y. (2011). Community dependence on non-timber forest products in Malinau, East Kalimantan: An ethnobotany analysis and its implications for forest conservation. Berk. Penelit. Hayati.

[60] Dlamini C.S., Geldenhuys C.J. (2009). The socioeconomic status of the non-timber forest product subsector in Swaziland. South. For..

[61] Maroyi A. (2018). *Syzygium Cordatum* Hochst. ex Krauss: An Overview of Its Ethnobotany, Phytochemistry and Pharmacological Properties. Molecules.

[62] Maroyi A. (2011). The gathering and consumption of wild edible plants in Nhema communal area, Midlands province, Zimbabwe. Ecol. Food Nutr..

[63] Tesfay A., Tewolde-Berhan S., Birhane E., Rannestad M.M., Gebretsadik A., Hailemichael G., Haile M., Gebrekirstos A. (2024). Edible indigenous fruit trees and shrubs in Tigray, Ethiopia. Trees For. People.

[64] Maroyi A., Semenya S.S. (2019). Medicinal uses, phytochemistry and pharmacological properties of *Elaeodendron transvaalense*. Nutrients.

[65] Akinnifesi F.K., Sileshi G., Ajayi O.C., Chirwa P.W., Mng’omba S., Chakeredza S., Nyoka B.I. (2008). Domestication and conservation of indigenous Miombo fruit trees for improving rural livelihoods in southern Africa. Biodiversity.

[66] Haokip S.W., Shankar K., Lalrinngheta J. (2020). Climate change and its impact on fruit crops. J. Pharmacogn. Phytochem..

[67] Raju C., Pazhanivelan S., Perianadar I.V., Kaliaperumal R., Sathyamoorthy N.K., Sendhilvel V. (2024). Climate change as an existential threat to tropical fruit crop production—A review. Agriculture.

[68] Seile B.P., Bareetseng S., Koitsiwe M.T., Aremu A.O. (2022). Indigenous knowledge on the uses, sustainability and conservation of African ginger (*Siphonochilus aethiopicus*) among two communities in Mpumalanga Province, South Africa. Diversity.

[69] Seyoum Y., Teketay D., Shumi G., Wodafirash M. (2015). Edible wild fruit trees and shrubs and their socioeconomic significance in Central Ethiopia. Ethnobot. Res. Appl..

[70] Burring J.H. (2004). *Canthium* *inerme*. PlantZAfrica.

